# HIV-1 Variants and Drug Resistance in Pregnant Women from Bata (Equatorial Guinea): 2012-2013

**DOI:** 10.1371/journal.pone.0165333

**Published:** 2016-10-31

**Authors:** Patricia Alvarez, Carolina Fernández McPhee, Luis Prieto, Leticia Martín, Jacinta Obiang, Pedro Avedillo, Antonio Vargas, Pablo Rojo, Agustín Benito, José Tomás Ramos, África Holguín

**Affiliations:** 1 HIV-1 Molecular Epidemiology Laboratory, Microbiology and Parasitology Department, Hospital Universitario Ramón y Cajal, IRYCIS and CIBERESP, Madrid, Spain; 2 Pediatrics Department, Hospital Universitario Gregorio Marañón, Madrid, Spain; 3 Pediatrics Department, Hospital Universitario de Getafe, Madrid, Spain; 4 Pediatrics Department, Hospital Regional de Bata, Ministerio de Sanidad y Bienestar Social, Bata, Equatorial Guinea; 5 Centro Nacional de Medicina Tropical, Instituto de Salud Carlos III-Madrid, RICET, Madrid, Spain; 6 Pediatrics Department, Hospital Universitario Doce de Octubre, Madrid, Spain; 7 Pediatrics Department, Hospital Universitario Clínico San Carlos, Madrid, Spain; George Washington University, UNITED STATES

## Abstract

**Objectives:**

This is the first study describing drug resistance mutations (DRM) and HIV-1 variants among infected pregnant women in Equatorial Guinea (GQ), a country with high (6.2%) and increasing HIV prevalence.

**Methods:**

Dried blood spots (DBS) were collected from November 2012 to December 2013 from 69 HIV-1 infected women participating in a prevention of mother-to-child transmission program in the Hospital Regional of Bata and Primary Health Care Centre *María Rafols*, Bata, GQ. The transmitted (TDR) or acquired (ADR) antiretroviral drug resistance mutations at partial *pol* sequence among *naive* or antiretroviral therapy (ART)-exposed women were defined following WHO or IAS USA 2015 lists, respectively. HIV-1 variants were identified by phylogenetic analyses.

**Results:**

A total of 38 of 69 HIV-1 specimens were successfully amplified and sequenced. Thirty (79%) belonged to ART-experienced women: 15 exposed to nucleoside reverse transcriptase inhibitors (NRTI) monotherapy, and 15 to combined ART (cART) as first regimen including two NRTI and one non-NRTI (NNRTI) or one protease inhibitor (PI). The TDR rate was only found for PI (3.4%). The ADR rate was 37.5% for NNRTI, 8.7% for NRTI and absent for PI or NRTI+NNRTI. HIV-1 group M non-B variants caused most (97.4%) infections, mainly (78.9%) recombinants: CRF02_AG (55.2%), CRF22_A101 (10.5%), subtype C (10.5%), unique recombinants (5.3%), and A3, D, F2, G, CRF06_cpx and CRF11_cpx (2.6% each).

**Conclusions:**

The high rate of ADR to retrotranscriptase inhibitors (mainly to NNRTIs) observed among pretreated pregnant women reinforces the importance of systematic DRM monitoring in GQ to reduce HIV-1 resistance transmission and to optimize first and second-line ART regimens when DRM are present.

## Introduction

Equatorial Guinea (GQ) is a small country located in West Central Africa between Cameroon and Gabon. HIV/AIDS is still a major cause of mortality and morbidity in GQ, and the most common routes of HIV transmission are heterosexual, followed by vertical transmission from mother to child and transfusions [1]. HIV prevalence is high (6.2%) and in 2015 more than half of the 27,000 people living with HIV in the country were women aged 15 onwards (http://www.unaids.org/en/regionscountries/countries/equatorialguinea). In the last decade, HIV prevalence has increased in the Equatoguinean population of 15 to 49 years of age (3.2% in 2004 and 6.2% in 2014 and in pregnant women (1.5% in 1997 and 10.1% in 2013) [1].

The use of antiretroviral therapy (ART) started on a regular basis in 2005 in GQ, although sporadic treatments, interrupted exposure to drugs, and high rates of loss to follow-up could have caused the appearance of resistance mutations, affecting the success of a future systematic antiretroviral program if the drug families causing resistance were still given to women carrying resistant viruses. The use of antiretroviral (ARV) regimens with a high genetic barrier to resistance, including new drug families, combined with improved patient adherence may mitigate acquired antiretroviral drug resistance mutations (ADR) rates, reducing the generation of new ARV-resistant strains. However, their higher prices are problematic for their wide use in poor countries with high HIV prevalence.

After confirmation of positive HIV diagnosis using 3 rapid tests according to GQ’s National Protocol [2], combined ART (cART) including zidovudine (AZT) + lamivudine (3TC) + nevirapine (NVP) are given to pregnant women with World Health Organization (WHO) clinical stage 3 or 4, or CD4 under 350 cell/mm^3^ as recommended by the National Plan to Combat HIV/AIDS in GQ. However those with WHO clinical stage 1 or 2, or CD4 above 350 cells/mm^3^ receive prophylaxis with AZT from the 28^th^ week of gestation, with AZT, 3TC and NVP at the time of delivery, followed by AZT and 3TC for one week postpartum to avoid the selection of resistance to single dose NVP. The protocol recommends that, after birth, the child receives a single dose of NVP and prophylaxis with AZT for 1 to 4 weeks.

Previous reports show that the rate of pregnant women who received antiretroviral therapy in GQ to prevent mother-to-child transmission (PMTCT) increased from 30.2% in 2012 to 78.6% in 2013. This is higher than in the general population which in 2013 increased 28.4% in adults and 6.3% in children [1]. Surveillance of drug resistance to antiretroviral drugs is particularly important in the pregnant population as infection by drug-resistant virus has implications for both maternal treatment and neonatal prophylaxis.

The WHO recommends population-based surveys to detect whether the prevalence of resistance in ART *naive* and ART-treated people is reaching alerting levels [3]. Updated resistance data can guide clinicians towards first-antiretroviral regimen selection in drug-*naive* subjects or to optimization of second-line ART regimens in pretreated patients under virological failure. However, despite its high and increasing HIV prevalence, the scale-up of ART in GQ, and the close location to the epicenter of the HIV-1 pandemic, the country lacks systematic surveillance studies to monitor drug resistance and to detect changes in HIV-1 molecular epidemiology. HIV-1 genotypic resistance testing is routinely done in developed countries to identify drug resistance mutations but it has not yet been implemented in GQ.

Identification of the circulating HIV-1 variants is important since some of them have clinical relevance, affecting disease progression [4]. Furthermore, due to high genetic variability and viral evolution, HIV presents variant-specific natural polymorphisms along the viral genome, some of them in residues associated with drug resistance [5,6]. They can influence the genetic barrier [7], the resistance pathways [8], the viral fitness [9], and the susceptibility to specific antiretrovirals [10,11]. Also, some natural polymorphisms favor the emergence of variants fully resistant to antiretrovirals [12], which not only affect drug-resistance interpretation when using different algorithms and generate discrepant results [13], but also increase ART-failure risk in some subtypes [14–16]. HIV-1 genetic variability can also lead to viral load (VL) underestimation or to no HIV-1 RNA detection [17], thus affecting early molecular diagnosis and ART-monitoring [17–20]. Since dried blood spots (DBS) are less infective, easier to collect, store, and transport than whole blood or plasma and with comparable performance for monitoring HIV infection in Public Health Programs [21], the WHO has recommended their use for improving access to virological testing, including viral load and drug resistance monitoring [22].

For a better efficacy of PMTCT regimens, documenting the profile of drug resistance in HIV-infected pregnant women is crucial for improving the maternal ART and prophylaxis in infants. To date, only few studies have reported resistance data and information regarding the HIV-1 subtypes and recombinants circulating in GQ. All the published data were obtained from specimens collected from 1997 to 2011 [23–25], but no data have been reported about the pregnant women collective. These previous studies revealed a complex epidemic in the country, where the prevalent HIV-1 variant is CRF02_AG, as in the neighboring countries [26]. Thus, the main objective of this study was to describe the presence of transmitted antiretroviral drug resistance mutations (TDR) and ADR and the current circulating HIV-1 variants among HIV-1 infected pregnant women participating in a PMTCT program at Hospital Regional of Bata and Primary Health Care Centre *María Rafols*, in Bata, GQ during the 2012–2013 period.

## Materials and Methods

### Study population and sample collection

The PMTCT program is a national and centralized program previously established in 2008 in GQ and centralized at two centers in Bata (Hospital Regional and Primary Health Care Center *Maria Rafols*) and one in Malabo (Hospital Regional). For this study, DBS were collected from all the 69 HIV-1 infected women following the PMTCT program during the 14 months study period (November 2012 to December 2013) in the two centers from Bata. All of them gave written informed consent to participate in this study, previously approved by human subjects review committees at Hospital Regional of Bata (Bata, GQ) and Hospital Universitario de Getafe (Madrid, Spain).

The samples were collected close to delivery by venipuncture and two drops of blood were spotted into each dot on a Whatman^™^ 903 Protein Saver Card (Schleicher & Schuell, USA). The women had a median age of 22 years (IQR 20–25). These dots, when saturated, are estimated to hold about 70 μl of blood. DBS cards were dried separately on a drying-rack overnight at room temperature in the hospital. Two cards (5 dots per card) were taken per patient, sealed in a zip-lock plastic bag with desiccant bags and stored at -20°C until their transport in dry ice to the laboratory in Madrid, Spain, where the samples were stored at -80°C until further use.

### HIV-1 sequencing for resistance testing and variant identification

RNA was extracted from DBS using the NucliSENS easyMAG automated platform (BioMèrieux, Marcy l´Etoile, France), followed by an in-house RT-PCR and nested-PCR to amplify the complete HIV-1 protease gene (codons 1–99) and partial reverse transcriptase gene (codons 1–335) as previously described [27]. PCR amplicons were purified using the illustra^™^ ExoProStar^™^ (GE Healthcare Life Sciences, Little Chalfont, UK) and sequenced by Macrogen Inc. (Gasan-dong, Geumchun-gu, Seoul, Korea).

For resistance testing, *pol* sequences (protease-PR and/or partial reverse transcriptase-RT) from antiretroviral-*naive* pregnant women were used to identify the TDR and quantify its prevalence following the WHO mutation list [28] and using the Calibrated Population Resistance tool (http://cpr.stanford.edu). ADR was studied using *pol* sequences from antiretroviral-exposed subjects considering substitutions following the 2015 International AIDS Society-USA (IAS-USA) mutation list [29].

In those viruses carrying DRM, predicted susceptibility to each drug was evaluated using Stanford’s HIVdb Algorithm (http://sierra2.stanford.edu/sierra/servlet/JSierra) in the available PR and/or RT sequence. We classified predicted drug resistance in three categories depending on mutation scores: low-level, intermediate, and high-level resistance according to Stanford’s HIVdb Algorithm.

For HIV-1 subtype or CRF characterization we performed phylogenetic analyses (phy) using the Phylip software after editing the generated *pol* sequences using SeqMan from the DNAstar package v7.1.0 (Lasergene, USA). We aligned sequences including at least two representative sequences of each 9 HIV group M subtypes and each 66 of the 79 circulating recombinant forms (CRFs) available at Los Alamos HIV sequence database (http://www.hiv.lanl.gov/content/sequence/HIV/CRFs/CRFs.html), as well as additional *pol* sequences from circulating HIV-1 variants previously described in GQ [25]. The tree topology was obtained using the Neighbour-Joining method. Alignment of DNA sequences was performed using the ClustalW program. The pairwise distance matrix was estimated using the Kimura two-parameter model. Bootstrap re-sampling (1000 data sets) of the multiple alignments was performed to test the statistical robustness of the tree, considering only the nodes with a bootstrap value over 700 [30]. Sequences not identified as any known subtype or CRF by phy were considered unique recombinant forms (URF).

### Accession Numbers

PR and/or RT sequences from DRM and subtyping testing of the study population were submitted to GenBank (www.ncbi.nlm.nih.gov/genbank) with the following accession numbers: KP890858-KP890927.

### Viral load quantification

We used the VERSANT^®^ HIV-1 RNA 1.0 (kPCR) assay (Siemens Healthcare Diagnostics, Tarrytown, USA) for HIV-1 plasmatic VL quantification from DBS. One spot was incubated for 30 minutes at room temperature with gentle rotation in 2 mL of Siemens DBS-specific lysis buffer including guanidine thiocyanate (not available for sale). Then, 1.1 mL of the supernatant was processed for automated nucleic acid extraction. Amplification software and settings of the kPCR assay were used without modification, as previously published [31]. As the haematocrit percentage was unknown in each pregnant woman, we assumed a mean value of 31.7% [32] in order to estimate the corrected DBS VL result, as recently reported [31]. The limit of detection of this assay is 894 HIV-1 RNA copies/mL (cp/mL) when using DBS [31].

### Statistical analysis

All statistical analyses were conducted using SPSS 13.0. Prevalences were expressed in percentage. Significance was set at p<0.05.

## Results

The 69 HIV-1 infected pregnant women selected during the PMTCT program in two health centers in Bata, GQ, during 2012–2013, had a median age of 22 years, all were infected by the heterosexual route, and half of them were asymptomatic (50.7% with WHO clinical stage 1) (Table 1). Among the 69 women, 12 (17.4%) were *naiv*e for the three drug classes at sampling time, 55 (79.7%) were on TAR and in 2 (2.9%) cases the presence or absence of ART was not reported in clinical reports. Among these 55 women, 21 had received monotherapy with AZT and 34 cART as first regimen. We were able to amplify HIV-1 partial *pol* sequences from 38 (55.1%) of the 69 infected pregnant women under study.

**Table 1:** Epidemiological and virological features of the HIV-1 infected study population.

Features	Study cohort	With available PR and/or RT sequence
Number of HIV-1 infected women	69	38
Htsex transmission route	69 (100%)	38 (100%)
Clinical status (WHO)		
I	35 (50.7%)	19 (50%)
II	2 (2.9%)	1 (2.6%)
III	1 (1.4%)	0
IV	0	0
Unknown	31 (44.9%)	18 (47.4%)
Vaginal delivery	55 (79.7%)	30 (78.9%)
HIV-1 transmission to newborns	2 (2.9%)	2 (5.3%)
ARV therapy		
ARV *naive* for the three drug families	12 (17.4%)	8 (21.1%)
ARV exposed^1^	55 (79.7%)	30 (78.9%)
Unknown	2 (2.9%)	0
Carrying DRM	-	8 (21.1%)
HIV-1 variants		
Subtype B	-	1 (2.6%)
Non-B subtypes	-	37 (97.4%)
Recombinants	-	29 (76.3%)

Htsex, heterosexual; WHO, World Health Organization; ARV, antiretroviral; DRM, drug resistance mutations. Among the 38 patients with available *pol* sequence data, 34 had available PR and 28 available RT sequences.

Among the 38 women with available *pol* sequence (34 had available PR and 28 available nearly complete RT sequences), 30 (78.9%) were under ART when samples were collected. The remaining 8 (21.1%) women were ARV *naive* for the three drug families (Table 1). However, when considering specific drug families, 8 of the 38 were *naive* for NRTI, 25 for NNRTI and 33 for PI. These *naive* women did not have ARV experience as they had not received previous antiretroviral therapies or any form of antenatal care according to clinical reports in GQ. S1 Table lists the main clinical and virological features of the 69 women under study, including age, type of delivery, viraemia, HIV-1 variant, ART experience prior to sampling time, and DRM found in each drug family. Among the 69 women of the study cohort, 35 (50.7%) had VLs >1,000 cp/ml (value considered as detectable viraemia according to WHO recommendations when using DBS), 13 (18.8%) had VLs <1,000 cp/ml and in 21 (30.4%) cases HIV-1 was not detected. In 3 (8.6%) of the 35 patients with VLs >1,000 cp/mL it was not possible to amplify the virus genome to obtain the sequence.

Considering only the 55 women under TAR at sampling time, 28 (50.9%) had VLs <1,000 cp/mL, and 27 (47.9%) had VLs >1,000 cp/mL. The high VL among 19 (76%) women under TAR under ART and VL>1,000 without MDR could suggest suboptimal adherence or unadequate dosing, among others. Unfortunately, information related to adherence to ART was not available in clinical reports.

Considering the 31 patients with no available sequence, 10 had VL <1,000 cp/mL, in 18 cases the virus was not detected and only 3 women had VL >1,000 cp/mL ranging 1,065–2,016 cp/mL. Although most (66.7%) of the 12 ARV-*naive* pregnant women presented detectable viraemia as expected in the absence of ART, 3 (25%) of them showed VL<1000 cp/ml and 1 had undetected vireamia (S1 Table). Among the 38 women with available *pol* sequence, 32 (84.2%) had VL>1,000 cp/ml.

Fifteen (50%) of the ART-treated women had been exposed to nucleoside reverse transcriptase inhibitors (NRTI), mainly to monotherapy with AZT. The other fifteen had received cART as first regimen, based on triple therapy including two NRTI [emtricitabine (FTC), tenofovir (TDF), AZT or 3TC] and one non-NRTI [NNRTI; efavirenz (EFV) or NVP] or one protease inhibitor or (PI)-boosted Lopinavir (LPV/r). The ARV experience in each woman is shown in S1 Table. Among the 30 ART-experienced pregnant women with available *pol* sequence, 25 (83.3%) presented VL >1,000cp/ml at sampling time (median: 2,405 cp/mL; range: 1,192–178,025).

S2 Table shows all mutations found at viruses from the 38 women with available PR and/or RT sequence. No HIVDR was observed in these 12 patients *naive* for the three drug families. Considering drug families, no TDR was found in NRTI or NNRTI-*naive* women, but the TDR rate for PI was 3.4% (1/29), since D30N change at PR was detected in a PI-*naive* woman carrying a CRF02_AG recombinant. The ADR rate was 37.5% for NNRTI, 8.7% for NRTI and was absent for PI among women exposed to the corresponding drug family (S2 Table). No case of NNRTI+NRTI resistance was found. When considering the 25 patients on ART at sampling time with VL>1,000 cp/ml and available sequence, 6 (24%) presented ADR. Table 2 shows the drug resistance rate and the DRM found among the 9 pregnant women from GQ carrying HIV-1 resistant variants. In the study cohort, none of the 9 women carrying resistance viruses transmitted the infection to their newborns and only 2 cases of HIV transmission to newborns occurred, representing a rate of HIV-1 transmission of 2.9% [33], lower than that reported in previous years [34].

**Table 2:** Epidemiological and virological features of the 9 pregnant women carrying resistant viruses in GQ (2012–2013).

ID	ART experience	ARV family (drug experience)			Viral load (log)	HIV-1 Variant	DRM			Predicted potential resistance level and affected drug^1^		
		NRTI	NNRTI	PI			to PI (in PR)	NRTI (in RT)	NNRTI (in RT)	High resistance	Moderate resistance	Low resistance
55	Monotherapy	AZT	-	-	ND	CRF02_AG	D30N*	NA	NA	NFV	-	-
39	Monotherapy	AZT	-	-	5.3	CRF02_AG	-	M41L	-	-	-	AZT, d4T,DDI
18	cART	TDF, FTC	EFV	-	4.3	CRF02_AG	-	M184V	-	FTC,3TC	-	DDI, ABC
34	cART	AZT, 3TC, FTC/TDF	NVP, EFV	LPV/r	3.9	Sub-subtype A3	-	-	V106I	-	-	-
20	cART	TDF, FTC	EFV	-	2.9	Subtype C	NA	-	V179D, G190A	NVP	EFV	ETR, RPV
15	cART	TDF, FTC	EFV	-	5.2	CRF02_AG	-	-	E138A	-	-	ETR, RPV
59	naive	none	none	none	2.9	CRF02_AG	-	-	V90I*	-	-	-
51	Monotherapy	AZT	-	-	4.5	CRF02_AG	-	-	V90I*	-	-	-
27	cART	AZT, 3TC	-	LPV/r	4.4	CRF02_AG	-	-	V90I*	-	-	-

ART, antiretroviral therapy; ARV, antiretroviral drug; DRM, drug resistance mutation according to IAS-USA 2015; NRTI, nucleoside reverse transcriptase inhibitors: AZT, zidovudine; d4T, stavudine; DDI, didanosine; ABC, abacavir; FTC, emtricitabine; 3TC, lamivudine; NNRTI, non-nucleoside reverse transcriptase inhibitors: NVP, nevirapine; EFV, efavirenz; ETR, etravirine; RPV, rilpivirine; PI, protease inhibitors: NFV, nelfinavir; cART, highly active antiretroviral therapy; NA, data not available due to unsuccessful PCR- amplification in that genome region; ND, not detected (<894 cp/mL according to Alvarez et al., 2014 [31]); Dash, no mutation or no predicted potential resistance found. Viral load in log RNA-HIV-1 copies/mL using Siemens VERSANT HIV-1 RNA 1.0 kPCR assay. With asterisk, mutations considered as transmitted drug resistant. All women were receiving those antiretrovirals at genotyping time.

* V90I was found in women naïve for NNRTI.

^1^ According to Stanford’s HIVdb Algorithm (http://sierra2.stanford.edu/sierra/servlet/JSierra).

Regarding the circulating HIV-1 variants, we observed a complex HIV-1 molecular epidemiology in infected pregnant women, in agreement with previous studies in patients from GQ [25]. Most (97.4%) infections were caused by group M non-B variants, and were mainly (78.9%) recombinants (Table 3). CRF02_AG caused slightly more than half (55.2%) of the infections, followed by CRF22_A101 (10.5%), subtype C (10.5%), unique recombinant forms (5.3%) and 2.6% of each sub-subtypes A3 and F2, subtypes B, D, and G, and recombinants CRF06_cpx and CRF11_cpx. We found an increase of infections caused by recombinant variants with respect to that reported in previous studies in GQ during 1997–2011 preformed at different sampling period and distinct HIV-infected collectives [23–25] (Table 3).

**Table 3:** HIV-1 group M variants reported in patients from GQ during 1997–2013.

HIV-1 M variant	Yebra et al., 2013^1^	This study	
Sampling year	1997–2011	2012–2013	
HIV-1 infected collective	General population	Pregnant women	
Number of patients	278	38	**(p-value)**
Sequenced gene	*pol*	*pol*	
Subtyping method	phy	phy	
Pure subtypes	122 (43.9%)	9 (23.7%)	<0.05
A	38 (13.7%)	1 A3 (2.6%)	NS
B	20 (7.2%)	1 (2.6%)	NS
C	16 (5.7%)	4 (10.5%)	NS
D	14 (5.0%)	1 (2.6%)	NS
F	11 (4.0%)	1 F2 (2.6%)	NS
G	15 (5.4%)	1 (2.6%)	NS
H	8 (2.9%)	0	NS
Recombinants	156 (56.1%)	29 (76.3%)	<0.05
CRF02_AG	133 (47.8%)	21 (55.2%)	NS
CRF06_cpx	4 (1.4%)	1 (2.6%)	NS
CRF09_cpx	1 (0.4%)	0	NS
CRF11_cpx	7 (2.5%)	1 (2.6%)	NS
CRF13_cpx	3 (1.1%)	0	NS
CRF18_cpx	1 (0.4%)	0	NS
CRF22_01A1	3 (1.1%)	4 (10.5%)	<0.05
URF	4 (1.4%)	2 (5.3%)	NS

GQ, Equatorial Guinea; CRF, circulating recombinant form; URF, unique recombinant form. Phy, phylogenetic analysis of HIV-1 sequences (*pol* or *env*). NS, not significant (p>0.05).

^1^According to Yebra et al., 2013 [25]. That study analyzes *pol* sequences from 195 HIV-infected Equatoguinean subjects attending Spanish clinics during 1997–2011 and 83 GenBank sequences sampled in GQ in 1997 and 2008 from military personnel in Malabo and general infected population in the country.

## Discussion

The WHO 2016 guidelines recommend providing lifelong ART to all HIV patients regardless of CD4 cell count but the monitoring of drug resistant HIV-1 is still a challenge to control the HIV/AIDS epidemic. It needs to be strengthened on a global scale, and pregnant women living with HIV should be considered one of the key target population [35]. Treating all HIV-infected pregnant and breastfeeding women serves three purposes: improving the mother’s health, preventing mother-to-child transmission of HIV, and preventing the viral transmission from the mother to a sexual partner [36]. The possible harm of offering lifelong ART includes the potential for cumulative drug toxicity and the possibility of poor adherence with long-term use, leading to the development of drug resistance, which should be periodically monitored.

The identification of drug resistance mutations is crucial to publish first and rescue-line ART guidelines at country level, as previously reported [35,37]. During the last years, different rates of TDR or ADR to reverse transcriptase inhibitors have been reported in pregnant women from some countries in Latin America [38–41], the Caribbean [42], and Sub-Saharan Africa [43–51]. In GQ ART was scaled up in 2005. In 2012 National guidelines for the PMTCT program in GQ recommended prophylaxis with AZT or cART in all HIV-infected pregnant women depending on their CD4 count and WHO clinical stage (WHO Option A) [2]. However, despite the scaling up of ART in the country (74% in 2014) (http://www.unaids.org/en/resources/presscentre/featurestories/2015/november/20151127_equatorialguinea) GQ lacks systematic surveillance studies for monitoring drug resistance in the general population, including HIV-1 infected pregnant women, which can have an impact on the HIV vertical transmission of resistant viruses.

According to the last National Report in GQ, during 2013 a total of 28.2% (10,384/36,800) of pregnant women in the country knew her positive or negative HIV status [1]. Since HIV-prevalence among pregnant women was 10.1% in the same year in GQ, it should represent around 1,000 HIV-infected pregnant women in 2013 [1]. Thus, during the study period, our study population would represent around 7% (69/1,000) of infected pregnant women in the country and 100% of HIV-infected women under the national PMTCT program in Bata, the second biggest city of GQ located in the continental area, with around a quarter of the nearly 1 million inhabitants living in the country [52].

In low resource countries the margin of long-term ARV treatment success is narrow because NNRTI-based regimens have a low genetic barrier to resistance. In fact, ART failure with a fixed-dose NRTI/NNRTI combination occurs in 10% to 30% of patients per year [53–55], and most patients with virological failure acquire NRTI and/or NNRTI resistance [55–57]. The first-line therapy in GQ includes two NRTI and one NNRTI leading to a good response with the proper adherence. However, if the woman has previously received NNRTIs in a non-continuous way, there is a risk of selection of resistance to NNRTIs. Among the studied pretreated pregnant women in GQ, we observed a high rate of NNRTI resistance present in more than a third of women with available sequence, probably explained by the use of NVP and EFV as part of most PMTCT regimens in our study cohort. The selection of resistance to NNRTIs in cART could be favored by the low genetic barrier of NNRTIs and by inadequate adherence [58,59].

Regarding NRTI, DRM selection to AZT in pregnant women with low HIV-1 viraemia and receiving monotherapy with AZT during pregnancy is uncommon [60], as seen in our study. Since DRM to PI were absent in the treated population, PI could be a good candidate to be included in rescue regimens in GQ.

We observed a low prevalence of TDR in the population, with only one *naive* woman carrying resistant viruses maybe transmitted by her sexual partner. However, TDR might be more prevalent given the ART interruptions and other extenuating factors that negatively affect adherence in GQ and other resource poor nations [61]. This TDR could be selected (or extinguished) by future antiretroviral exposure in those women.

Our data reveal that 17.4% of the women participating in this study were *naive* for NRTI, NNRTI and PI, despite being included in a PMTCT program. Moreover, half of the 55 women on ART presented detectable viraemia at sampling time, suggesting low adherence to ART. The high VL observed at sampling time in the absence of DRM after ARV exposure among some treated women is also suggestive of an inappropriate adherence to treatment. Poor adherence to ART in pregnant women is a major problem in Sub-Saharan Africa, since it is a predictor of virological failure, emergence of resistant viruses, disease progression and death [35]. Long-standing health-system issues (such as staffing and service accessibility), educational factors (poor knowledge of HIV/ART/vertical transmission and a low maternal educational level) and community-level factors (particularly stigma, psychosocial distress, fear of disclosure and lack of partner or community support) still make the access to ART very difficult and could explain the lack of uptake of antiretroviral drugs in these women, as previously reported [61]. Our study encourages the importance of reinforcing the PMTCT program in Bata so that all of the participants receive ART before the delivery, avoiding losses in the cascade of care from HIV diagnosis to the start of ART.

Our results provide the most recent data regarding the HIV-1 molecular epidemiology in GQ. We confirmed the predominance of HIV-1 recombinant variants, observing CRF02_AG in nearly 55% of infections. We also noticed an increase of infections caused by URF compared to previous years. HIV-1 recombination is a powerful evolutionary force that occurs ten-fold more frequently than punctual mutations and produces and maintains further variability by shuffling and combining viral genetic material. The continuous evolution and diversification of HIV will bring new challenges for its prevention, treatment and virological monitoring using molecular tools [62–65]. As we did not study the complete HIV genome, the rate of recombinants could have been even higher if more viral regions had been assessed.

This study presents some limitations. Data on number of pregnancies or previously infected/exposed children from the 69 women were not available in clinical reports provided by GQ clinicians, but it is unlikely that these women were exposed to ART before participating in the PMTCT program. As most of the included 69 women were under ART at sampling leading to low VL, we were able to obtain resistance data from 38 women, representing 55.1% of the whole cohort, a high rate compared to other reports performed on treated pregnant women [38,48,49]. Following WHO recommendations for resistance testing in limited resources settings, we used DBS for sample collection (only four drops of eluted dried blood) to obtain the HIV sequence, whereas all resistance studies in pregnant women apart from one in Tanzania [43], were carried out on higher volumes of plasma or whole blood [38–42,44–51]. Although DBS samples were collected during 2012 and 2013, the drug resistance and molecular epidemiology data provided are the first to be reported in the pregnant women collective in GQ, giving valuable information for clinicians and for National Health authorities in the country.

The present data have relevant importance since, according to UNAIDS, correct HIV-1 monitoring and ART in Bata and Malabo, the two main cities in the country, would significantly reduce HIV infection in the country (http://www.unaids.org/es/resources/presscentre/featurestories/2015/november/20151127_equatorialguinea). Since the Bata population presents a lower HIV rate among pregnant women than the population of the insular region of GQ [1] and that the urban population in Bata presents large differences from the rural areas in GQ in terms of fertility rates and access to the current PTCTM services, resistance studies should be reinforced in this key collective of HIV-1 pregnant women in Equatorial Guinea.

In conclusion, the low presence of DRM to PI among *naive* women and the high DRM rate to retrotranscriptase inhibitors (mainly NNRTIs) observed among pretreated women, reinforce the importance of systematic DRM monitoring in pregnant women in GQ. This could reduce HIV-1 resistance transmission to newborns or to sexual partners, optimize first and second-line ART regimens when DRM are present, and preserve future ART options in infected patients carrying resistant viruses in the country. Continuous adherence counselling and periodic monitoring of the virological response is crucial for a successful implementation of ART in pregnant women during PMTCT programs.

## Supporting Information

S1 Table Clinical features, HIV-1 variant, and viral load of the 69 pregnant women under study in Equatorial Guinea. ID, identification; WHO, World Health Organization; PMTCT ART, antirretroviral therapy recieved during the prevention of mother-to-child transmission program; ART, antiretroviral; cp/ml HIV RNA copies/milliliter; DRM, drug resistance mutation; NRTI, nucleoside reverse transcriptase inhibitor; NNRTI, non-nucleoside reverse transcriptase inhibitor; PI, protease inhibitor; cART, combined antiretroviral therapy; ATZ, zidovudine; ND, not detected by kPCR assay. No, patients under ART including NRTI, NNRTI and PI. Patients highlighted in grey had detectable viral load (>1,000 cp/ml).
(DOC)

S2 Table Genotyping results among the 38 HIV-1 infected pregnant women with available PR and/or RT sequences. HIVDR, viral resistance mutations to antiretrovirals. In blue, changes at RT residues involved at resistance but not included at IAS2015 and Bennett list. With asterisk, changes present in virus collected from *naive* pregnant women absent in Bennett list for TDR but included in IAS2015 lists.
(DOCX)
