# Supplementary material for: HIV-1 Variants and Drug Resistance in Pregnant Women from Bata (Equatorial Guinea): 2012-2013
Source: PLoS One. 2016 Oct 31;11(10):e0165333. doi: 10.1371/journal.pone.0165333 (PMC5087953; doi:10.1371/journal.pone.0165333)
Supplement: S1 Table — ID, identification; WHO, World Health Organization; PMTCT ART, antirretroviral therapy recieved during the prevention of mother-to-child transmission program; ART, antiretroviral; cp/ml HIV RNA copies/milliliter; DRM, drug resistance mutation; NRTI, nucleoside reverse transcriptase inhibitor; NNRTI, non-nucleoside reverse transcriptase inhibitor; PI, protease inhibitor; cART, combined antiretroviral therapy; ATZ, zidovudine; ND, not detected by kPCR assay. No, patients under ART including NRTI, NNRTI and PI. Patients highlighted in grey had detectable viral load (>1,000 cp/ml). (DOC) [file pone.0165333.s001.doc]

**S1 Table.** Clinical features, HIV-1 variant, and viral load of the 69 pregnant women under study in Equatorial Guinea

| **ID** | **WHO clinical stage** | **Delivery** | **PMTCT ART** | **ART regimen** | **Naive to ARV** | **HIV-1 variant** | **Corrected Viral Load (cp/ml)** | **Corrected Viral Load (log10 cp/ml)** | **Available PR and/or RT sequence** |
| --- | --- | --- | --- | --- | --- | --- | --- | --- | --- |
| 1 | Unknown | Vaginal | cART | cART | No | Not amplified | ND | ND | No |
| 2 | Unknown | Vaginal | cART | cART | Naive to PI | CRF02_AG | 37,440 | 4.57 | Yes |
| 3 | Unknown | C-section | cART | cART | No | CRF02_AG | ND | ND | Yes |
| 4 | Unknown | C-section | cART | cART | Naive to NNRTI | G | 2,907 | 3.46 | Yes |
| 5 | Unknown | Vaginal | cART | cART | Naive to NNRTI | Not amplified | ND | ND | No |
| 6 | Unknown | Vaginal | cART | cART | Naive to NNRTI | Not amplified | 774 | 2,89 | No |
| 7 | I | Vaginal | cART | cART | Naive to PI | CRF02_AG | ND | ND | Yes |
| 8 | III | Vaginal | cART | cART | No | Not amplified | ND | ND | No |
| 9 | Unknown | Vaginal | cART | cART | Naive to PI | B | 774 | 2.89 | Yes |
| 10 | Unknown | Vaginal | cART | cART | Unknown | Not amplified | 774 | 2.89 | No |
| 11 | Unknown | Vaginal | cART | cART | Naive to PI | F2 | 1,723 | 3.24 | Yes |
| 12 | Unknown | Vaginal | cART | cART | Naive to PI | Not amplified | ND | ND | No |
| 13 | Unknown | Vaginal | cART | cART | No | Not amplified | 774 | 2.89 | No |
| 14 | Unknown | Vaginal | cART | cART | Naive to PI | CRF02_AG | 10,437 | 4.02 | Yes |
| 15 | Unknown | Vaginal | cART | cART | Naive to PI | CRF02_AG | 158,753 | 5.20 | Yes |
| 16 | Unknown | Vaginal | cART | cART | Naive to PI | Not amplified | ND | ND | No |
| 17 | Unknown | Vaginal | cART | cART | Naive to PI | Not amplified | ND | ND | No |
| 18 | Unknown | Vaginal | cART | cART | Naive to PI | CRF02_AG | 17,803 | 4.25 | Yes |
| 19 | Unknown | Vaginal | cART | cART | Naive to NNRTI and PI | Not amplified | ND | ND | No |
| 20 | Unknown | Vaginal | cART | cART | Naive to PI | C | 774 | 2.89 | Yes |
| 21 | I | Vaginal | cART | cART | Naive to PI | Not amplified | 1,640 | 3.21 | No |
| 22 | I | Vaginal | cART | cART | No | CRF02_AG | 3,033 | 3.48 | Yes |
| 23 | I | Vaginal | cART | cART | Naive to PI and NNRTI | Not amplified | ND | ND | No |
| 24 | I | C-section | cART | cART | Naive to PI | Not amplified | ND | ND | No |
| 25 | I | Vaginal | cART | cART | No | Not amplified | ND | ND | No |
| 26 | I | Vaginal | cART | cART | Naive to PI | Not amplified | 774 | 2.89 | No |
| 27 | I | Vaginal | cART | cART | Naive to NNRTI | CRF02_AG | 52,709 | 4.72 | Yes |
| 28 | I | C-section | cART | cART | Naive to PI | CRF02_AG | 1,324 | 3.12 | Yes |
| 29 | I | Vaginal | cART | cART | Naive to PI | Not amplified | ND | ND | No |
| 30 | II | Vaginal | cART | cART | Naive to PI | Not amplified | ND | ND | No |
| **ID** | **WHO clinical stage** | **Delivery** | **PMTCT ART** | **ART regimen** | **Naive to ARV** | **HIV-1 variant** | **Corrected Viral Load (cp/ml)** | **Corrected Viral Load (log10 cp/ml)** | **Available PR and/or RT sequence** |
| 31 | I | Vaginal | cART | cART | Naive to PI | CRF11_cpx | 3,179 | 3.50 | Yes |
| 32 | I | Vaginal | cART | cART | No | Not amplified | ND | ND | No |
| 33 | I | Vaginal | cART | cART | Naive to PI | Not amplified | ND | ND | No |
| 34 | Unknown | C-section | cART | cART | No | A3 | 7,132 | 3.85 | Yes |
| 35 | I | C-section | AZT | Monotherapy | Naive to NNRTI and PI | CRF22_01A1 | 22,380 | 4.35 | Yes |
| 36 | Unknown | Vaginal | AZT | Monotherapy | Naive to NNRTI and PI | CRF02_AG | 6,024 | 3.78 | Yes |
| 37 | Unknown | Vaginal | AZT | Monotherapy | Naive to NNRTI and PI | CRF02_AG | 1,192 | 3.08 | Yes |
| 38 | Unknown | Vaginal | AZT | Monotherapy | Naive to NNRTI and PI | CRF02_AG | 2,761 | 3.44 | Yes |
| 39 | Unknown | Vaginal | AZT | Monotherapy | Naive to NNRTI and PI | CRF02_AG | 178,025 | 5.25 | Yes |
| 40 | Unknown | Vaginal | AZT | Monotherapy | Naive to NNRTI and PI | CRF02_AG | 95,605 | 4.98 | Yes |
| 41 | Unknown | Vaginal | AZT | Monotherapy | Naive to NNRTI and PI | Not amplified | 833 | 2.92 | No |
| 42 | I | C-section | AZT | Monotherapy | Naive to NNRTI and PI | CRF22_01A1 | 1,661 | 3.22 | Yes |
| 43 | I | Vaginal | AZT | Monotherapy | Naive to NNRTI and PI | Not amplified | ND | ND | No |
| 44 | I | Vaginal | AZT | Monotherapy | Naive to NNRTI and PI | Not amplified | 774 | 2.89 | No |
| 45 | I | C-section | AZT | Monotherapy | Naive to NNRTI and PI | Not amplified | 885 | 2.95 | No |
| 46 | I | C-section | AZT | Monotherapy | Naive to NNRTI and PI | Not amplified | 2,016 | 3.30 | No |
| 47 | I | Vaginal | AZT | Monotherapy | Naive to NNRTI and PI | CRF02_AG | 4,957 | 3.70 | Yes |
| 48 | I | Vaginal | AZT | Monotherapy | Naive to NNRTI and PI | CRF02_AG | 1,707 | 3.23 | Yes |
| 49 | I | Vaginal | AZT | Monotherapy | Naive to NNRTI and PI | Not amplified | 774 | 2.89 | No |
| 50 | I | C-section | AZT | Monotherapy | Naive to NNRTI and PI | C | 3,075 | 3.49 | Yes |
| 51 | I | Vaginal | AZT | Monotherapy | Naive to NNRTI and PI | CRF02_AG | 26,354 | 4.42 | Yes |
| 52 | I | Vaginal | AZT | Monotherapy | Naive to NNRTI and PI | CRF22_01A1 | 1,391 | 3.14 | Yes |
| **ID** | **WHO clinical stage** | **Delivery** | **PMTCT ART** | **ART regimen** | **Naive to ARV** | **HIV-1 variant** | **Corrected Viral Load (cp/ml)** | **Corrected Viral Load (log10 cp/ml)** | **Available PR and/or RT sequence** |
| 53 | II | Vaginal | AZT | Monotherapy | Naive to NNRTI and PI | CRF06_cpx | 3,911 | 3.59 | Yes |
| 54 | I | Vaginal | AZT | Monotherapy | Naive to NNRTI and PI | URF | 2,405 | 3.38 | Yes |
| 55 | I | Vaginal | AZT | Monotherapy | Naive to NNRTI and PI | CRF02_AG | ND | ND | Yes |
| 56 | Unknown | Vaginal | None | Naive | Naive | C | 1,148 | 3.06 | Yes |
| 57 | Unknown | Vaginal | None | Naive | Naive | C | 2,280 | 3.36 | Yes |
| 58 | Unknown | Vaginal | None | Naive | Naive | Not amplified | ND | ND | No |
| 59 | I | Vaginal | None | Naive | Naive | CRF02_AG | 774 | 2.89 | Yes |
| 60 | Unknown | C-section | None | Naive | Naive | URF | 42,878 | 4.63 | Yes |
| 61 | I | C-section | None | Naive | Naive | Not amplified | 774 | 2.89 | No |
| 62 | I | Vaginal | None | Naive | Naive | Not amplified | 1,065 | 3.03 | No |
| 63 | Unknown | C-section | None | Naive | Naive | Not amplified | 774 | 2.89 | No |
| 64 | I | Vaginal | None | Naive | Naive | CRF02_AG | 15,603 | 4.19 | Yes |
| 65 | I | Vaginal | None | Naive | Naive | CRF02_AG | 2,364 | 3.37 | Yes |
| 66 | I | Vaginal | None | Naive | Naive | D | 3,054 | 3.48 | Yes |
| 67 | I | Vaginal | None | Naive | Naive | CRF22_01A1 | 1,272 | 3.10 | Yes |
| 68 | I | Vaginal | Unknown | Unknown | Unknown | Not amplified | ND | ND | No |
| 69 | Unknown | Unknown | Unknown | Unknown | Unknown | Not amplified | ND | ND | No |

ID, identification; WHO, World Health Organization; PMTCT ART, antirretroviral therapy recieved during the prevention of mother-to-child transmission program; ART, antiretroviral; cp/ml HIV RNA copies/milliliter; DRM, drug resistance mutation; NRTI, nucleoside reverse transcriptase inhibitor; NNRTI, non-nucleoside reverse transcriptase inhibitor; PI, protease inhibitor; cART, combined antiretroviral therapy; ATZ, zidovudine; ND, not detected by kPCR assay. No, patients under ART including NRTI, NNRTI and PI.

Patients highlighted in grey had detectable viral load (>1,000 cp/ml).
