# Supplementary material for: HIV-1 Variants and Drug Resistance in Pregnant Women from Bata (Equatorial Guinea): 2012-2013
Source: PLoS One. 2016 Oct 31;11(10):e0165333. doi: 10.1371/journal.pone.0165333 (PMC5087953; doi:10.1371/journal.pone.0165333)
Supplement: S2 Table — In blue, changes at RT residues involved at resistance but not included at IAS2015 and Bennett list. With asterisk, changes present in virus collected from naive pregnant women absent in Bennett list for TDR but included in IAS2015 lists. (DOCX) [file pone.0165333.s002.docx]

**S2 Table**. Genotyping results among the 38 HIV-1 infected pregnant women with available PR and/or RT sequences.

| **ID paper** | **ART regimen** | **Drug *naive* to** | | | **HIV-1 variant** | **HIVDR** | | | **RT** | **PR** |
| --- | --- | --- | --- | --- | --- | --- | --- | --- | --- | --- |
|  |  | **NRTI** | **NNRTI** | **PI** |  | **To NRTI** | **To NNRTI** | **To PI** |  |  |
| 2 | cART | No | No | Yes | CRF02_AG | None | None | None | D123E, I135L, I142K, S162A, T165I, F171Y, K173A, Q174K, D177E, T200A, Q207K, R211E, V245Q, K277R, L283I, T286A, E291D, V292T, I293V, L295M, K311R, S322T, I326V, G335D | Q2H, I3V, I13V, K14R, K20I, E35D, M36I, R41K, L63P, H69K, L89M |
| 3 | cART | No | No | No | CRF02_AG | - | - | None | Negative PCR | G16E, K20I, E35D, M36I, R41K, H69K, K70R, L89M |
| 4 | cART | No | Yes | No | G | None | None | None | E53D, I135V, S162A, K173I, Q174K, D177E, I178V, Q207D, R211K, V245Q | I13A, I15V, K20I, E35K, M36L, N37D, R41K, L63P, H69K, K70R, L89I |
| 7 | cART | No | No | Yes | CRF02_AG | - | - | None | Negative PCR | I13V, K14R, K20I, G27E, E35D, M36I, R41K, H69K, L89M |
| 9 | cART | No | No | Yes | B | - | - | None | I31V, S68G  Incomplete RT sequence | T12K, K14R, I15V, E35D, N37E, L63T, H69Y |
| 11 | cART | No | No | Yes | F2 | None | None | None | S162C, K173A, Q174K, D177E, I178L, Q207G, R211K, V245Q, D250T, A272P, K275R, Q278Y, T286A, V292I, I293V, E297A, D324E, Q334H, G335D | I15V, K20R, E35D, M36I, N37E, R41K, K45R, D60E, L63P, H69R, V75I, L89M, I93V |
| 14 | cART | No | No | Yes | CRF02_AG | None | None | None | V35A, K49R, V60I, S162A, K173A, Q174K, D177E, I195L, T200A, Q207E, P243A, V245K, T286A, E291D, V292I, I293V, P294T, S322T, G335D | T12P, I13V, K14R, I15V, G17E, L19I, K20I, M36I, L63P, H69K, P79D, L89M |
| 15 | cART | No | No | Yes | CRF02_AG | None | **E138A** | None | V35T, E40D, A98S, K122E, D123T, K122E, D123N, I135V, S162A, K173I, Q174K, D177E, I178V, T200A, Q207E, R211K, F214L, V245M, A272P, E291D, V292I, I293V, N306D, S322T, I326V | L10V, I13V, G16E, L19P, K20I, E35D, M36I, R41K, L63I, I64M, H69K, L89M |

| **ID paper** | **ART regimen** | **Drug *naive* to** | | | **HIV-1 variant** | **HIVDR** | | | **RT** | **PR** |
| --- | --- | --- | --- | --- | --- | --- | --- | --- | --- | --- |
|  |  | **NRTI** | **NNRTI** | **PI** |  | **To NRTI** | **To NNRTI** | **To PI** |  |  |
| 18 | cART | No | No | Yes | CRF02_AG | **M184V** | None | None | P4S, E6D, V35K, E36D, S48T, K49R, V60I, A98S, K122P, I135V, S162A, E169D, K173T, Q174T, N175Y, D177E, T200A, Q207E, R211I, V245Q, K249Q, T286A, E291D, V292I, I293V, P294T, K311R, E312D, I326V | L10V, I13V, G16E, K20I, M36I, N37D, R41K, L63S, H69K, L89M |
| 20 | cART | No | No | Yes | C | None | **V179D, G190A** | - | I135V, S162C, K173T, Q174R, D177E, T200A, Q207E, R211K, V245Q, E248D, A272P, K277R, T286A, E291D, V292I, I293V, I326V, Q334N, G335D | Negative PCR |
| 22 | cART | No | No | No | CRF02_AG | - | - | None | Negative PCR | I13V, K20I, E35K, M36I, R41N, H69K, K70R, L89M |
| 27 | cART | No | Yes | No | CRF02_AG | None | None | None | **V90I***, D123E, I135V, S162A, K173T, Q174E, D177E, T200A, Q207T, R211K, V245K, A272P, K281R, T286A, E291D, V292I, I293V, S322T, I326V, G335D | I13V, K20I, E35D, M36I, N37D, R41K, L63P, H69K, T74S, L89M |
| 28 | cART | No | No | Yes | CRF02_AG | - | - | None | Negative PCR | I13V, G16E, K20I, E35D, M36I, R41K, H69K, K70R, L89M |
| 31 | cART | No | No | Yes | CRF11_cpx | None | None | None | I5V, E6D, V35I, S68G, K122P, D123S, I135T, E169D, K173T, D177E, I178L, T200E, I202V, Q207K, R211K, V245Q, E248D, D250E, A272P, K277R, E291D, V292I, I293V, P313T, S322T, I326V, I329V, Q334L, G335D | T12K, G16A, K20R, E35G, M36I, N37D, R41K, D60E, Q61E, I62V, L63I, C67E, H69K, V77I, L89M |
| 34 | cART | No | No | No | A3 | None | **V106I** | None | K11T, V35T, E36A, T39K, K43E, **K103Q**,  K104R, , D123N, K173L, D177E, I178V, V179I, T200A, Q207A, R211K, F214L, V245Q, E248N, D250E, T286A, E291D, V292I, I293V, P294T, E312D, I326V, G335D | L10V, I13V, K14R, M36I, R41K, L63P, H69K, L89M |
| 35 | Monotherapy | No | Yes | Yes | CRF22_01A1 | None | None | None | V60I, D121Y, K122E, I135T, E169D, K173T, Q174K, D177E, G196E, Q197H, T200A, Q207A, R211S, I244V, E248N, A272P, L283I, E291D, V292I, I293V, V317A | L10V, I13V, E35D, M36I, N37D, R41K, I62V, L63A, H69K, K70R, T74S, L89M |
| 36 | Monotherapy | No | Yes | Yes | CRF02_AG | None | None | - | D121Y, K122E, S162A, K173T, Q174K, D177E, I202V, Q207K, R211E, V245Q, T286A, E291D, V292I, I293V, P294T | Negative PCR |

| **ID paper** | **ART regimen** | **Drug *naive* to** | | | **HIV-1 variant** | **HIVDR** | | | **RT** | **PR** |
| --- | --- | --- | --- | --- | --- | --- | --- | --- | --- | --- |
|  |  | **NRTI** | **NNRTI** | **PI** |  | **To NRTI** | **To NNRTI** | **To PI** |  |  |
| 37 | Monotherapy | No | Yes | Yes | CRF02_AG | None | None | None | K20R,V35T, V60T, I135V, S162A, K173T, Q174H, D177E, T200A, Q207E, F214L, V245Q, K281R, T286A, E291D, V292I, I293V, P294V, S322A, I326V | K20I, M36I, N37E, R41K, K43R, Q61H, L63P, H69K, L89M |
| 38 | Monotherapy | No | Yes | Yes | CRF02_AG | None | None | - | K122E, D123S, I135V, S162A, K173A, Q174K, D177E, Q207A, R211K, V245Q, E248D, T286A, E291D, V292I, I293V, P294A, S322A, I326V, G335D | Negative PCR |
| 39 | Monotherapy | No | Yes | Yes | CRF02_AG | **M41L** | None | None | I5N, E28A, K32KR, V35T, E36D, I135V, S162A, S163T, T165I, E169D, K173T, Q174K, D177E, I178M, T200A, Q207E, R211K, V245Q, E248D, S251N, K275Q, V276T, K281R, T286A, E291D, V292I, I293V, P294T, S322T, I326V, Q330R | I13V, G16GE, L19V, K20I, E35D, M36L, N37H, R41K, L63P, H69K, K70KR, L89M |
| 40 | Monotherapy | No | Yes | Yes | CRF02_AG | None | None | None | E28A, V35T, E36D, T107S, K122E, I135V, S162A, E169D, K173T, Q174K, D177E, T200A, Q207E, R211K, V245Q, E248D, S251N, V276T, T286A, E291D, V292I, I293V, E297K, A304E, S322T, I326V, G335D | I13V, L19V, K20I, E35D, M36L, N37H, R41K, H69K, L89M |
| 42 | Monotherapy | No | Yes | Yes | CRF22_01A1 | None | None | None | K122E, D123G, E169D, K173A, Q174K, D177E, G196E, Q207A, R211S, V245T, A272P, V276I, T286A, E291D, I293V, E312D, S322L, I326V, G335D | I13V, E35N, M36I, N37D, R41K, H69K, I72L, L89M |
| 47 | Monotherapy | No | Yes | Yes | CRF02_AG | None | None | None | V35T, T39G, D123E, I135V, S162A, K173I, Q174K, D177E, T200A, Q207A, R211K, F214L, V245L, A272P, K281R, T286A, E291D, V292I, I293V, K311R, S322A, I326V, G335D | I13V, G16E, K20I, E35D, M36I, R41K, L63S, H69K, K70R, L89M |
| 48 | Monotherapy | No | Yes | Yes | CRF02_AG | None | None | None | V35T, E36D, T39S, I135V, S162A, F171Y, K173A, Q174K, D177E, T200A, Q207E, K238R, K275R, V276I, K281R, E291D, I293V, P294T, E312N, I326L | I13V, I15V, K20I, M36I, R41K, I64M, H69K, L89M |
| 50 | Monotherapy | No | Yes | Yes | C | None | None | None | I135V, S162C, K173T, Q174R, D177E, T200A, Q207E, R211K, V245Q, E248D, A272P, K277R, T286A, E291D, V292I, I293V, I326V | I15V, L19V, E35D, M36I, N37S, R41K, D60E, L63T, H69K, L89M, I93L |

| **ID paper** | **ART regimen** | **Drug *naive* to** | | | **HIV-1 variant** | **HIVDR** | | | **RT** | **PR** |
| --- | --- | --- | --- | --- | --- | --- | --- | --- | --- | --- |
|  |  | **NRTI** | **NNRTI** | **PI** |  | **To NRTI** | **To NNRTI** | **To PI** |  |  |
| 51 | Monotherapy | No | Yes | Yes | CRF02_AG | None | **None** | None | W24C, V35T, **V90I*,** D123E, I135V, S162A, Q174E, D177E, T200A, Q207K, V245Q, A272P, T286A, E291D, V292I, I293V | V11I, I13V, K14R, K20I, E35D, M36I, R41K, L63P, H69K, L89M |
| 52 | Monotherapy | No | Yes | Yes | CRF22_01A1 | - | - | None | Negative PCR | T12R, I13V, G16A, L19I, E35D, M36I, N37D, R41K, K43R, H69K, L89M |
| 53 | Monotherapy | No | Yes | Yes | CRF06_cpx | None | None | None | V21I, V35T, V60I, K122Q, S162A, K173A, Q174N, D177E, T200A, Q207A, V245Q, E248D, A272P, K277R, T286A, A288T, V292I, I293V, E297A, G335D | I13V, K14R, I15V, K20I, E35D, M36I, R41K, L63T, I64M, H69K, L89M |
| 54 | Monotherapy | No | Yes | Yes | URF | None | **None** | None | **K103E** , K122E, D123S, I135T, S162H, K173S, D177E, V179I, Q207A, R211K, F214L, V245Q, E248N, D250E, S251C, A272P, K277R, T286A, E291D, I293V, E312D, I326V | L10I, I13V, G16E, K20I, E35K, M36I, N37E, R41K, K43R, R57K, L63V, C67S, H69R, L89M |
| 55 | Monotherapy | No | Yes | Yes | CRF02_AG | - | - | **D30N** | E6A, V35T, E36D, T39L, M41I, G45E, S48T, A62T incomplete RT sequence | K20I, M36I, N37D, R41K, H69K, L89M |
| 56 | *Naive* | Yes | Yes | Yes | C | None | None | - | V60I, I135V, K173T, Q174R, D177E, T200A, Q207E, R211K, V245Q, E248N, A272P, K277R, T286A, E291D, V292I, I293V, I326V, Q334N, G335D | Negative PCR |
| 57 | *Naive* | Yes | Yes | Yes | C | - | - | None | V35T, E36A, T39E, S48T, V60I  incomplete RT sequence | T12A, I15V, L19V, M36I, R41K, L63V, H69K, L89M, I93L |
| 59 | *Naive* | Yes | Yes | Yes | CRF02_AG | None | None | None | **V90I*,** A98S, S162A, K173I, Q174K, T200A, Q207E, R211K, V245Q, D250E, T286A, E291D, V292I, I293V, P294V, S322T, I326V, G335D | I13V, K20I, E35D, M36I, R41K, L63S, H69K, K70R, L89M |
| 60 | *Naive* | Yes | Yes | Yes | URF | None | None | None | V35T, T39K, K43E, K49R, K64R, D121Y, K122E, I135T, T165I, E169D, K173A, Q174K, D177E, Q207A, R211K, V245N, E291D, V292I, I293V, K311R, E312N | I13V, G16E, K20R, E35D, M36I, N37K, R41K, H69K, K70R, L89M |

| **ID paper** | **ART regimen** | **Drug *naive* to** | | | **HIV-1 variant** | **HIVDR** | | | **RT** | **PR** |
| --- | --- | --- | --- | --- | --- | --- | --- | --- | --- | --- |
|  |  | **NRTI** | **NNRTI** | **PI** |  | **To NRTI** | **To NNRTI** | **To PI** |  |  |
| 64 | *Naive* | Yes | Yes | Yes | CRF02_AG | None | None | None | D123E, S162A, K173A, Q174A, D177E, T200A, Q207E, F214L, V245Q, D250E, S251D, A272P, E291D, V292I, I293V, L310I, S322T, I326V, G335D | I13V, K20I, E35D, M36I, R41K, L63S, I64M, H69K, L89M |
| 65 | *Naive* | Yes | Yes | Yes | CRF02_AG | None | None | None | V35K, E36D, S48T, K49R, V60I, A98S, I135V, S162A, K173T, Q174K, D177E, T200A, Q207E, K238R, V245Q, T286A, E291D, V292I, I293V, P294T, K311R, E312D, I326V, G335D | K20I, M36I, N37D, R41K, L63P, H69K, K70R |
| 66 | *Naive* | Yes | Yes | Yes | D | - | - | None | K32I, V35T, K49R, V60I, R83K, V118I incomplete RT sequence | L10V, I13V, I15V, K20V, M36I, N37D, K43R, R57K, L63S, I64V |
| 67 | *Naive* | Yes | Yes | Yes | CRF22_01A1 | - | - | None | I5V, K11A, E28K, K32E, V35T, T39K, V60I incomplete RT sequence | T12S, I13V, K20R, E35D, M36I, N37D, R41K, H69K, L89M |

HIVDR, viral resistance mutations to antiretrovirals. In blue, changes at RT residues involved at resistance but not included at IAS2015 and Bennett list. With asterisk, changes present in virus collected from *naive* pregnant women absent in Bennett list for TDR but included in IAS2015 lists.
